# The Impact of Dementia Caregiving on Sleep and Depression: The Role of Social Support and Rural-Urban Differences

**DOI:** 10.1093/geroni/igaf122.2984

**Published:** 2025-12-31

**Authors:** Heehyul Moon, Sunshine Rote, Ann Folker, Kathryn Williams Sites

**Affiliations:** University of Louisville, Louisville, Kentucky, United States; University of Louisville, Louisville, Kentucky, United States; University of Massachusetts Amherst, Amherst, Massachusetts, United States; University of Louisville, Louisville, Kentucky, United States

## Abstract

Dementia caregivers experience greater psychological distress, including sleep disturbances and depression, compared to non-dementia caregivers. Poor sleep is a key predictor of depressive symptoms, yet the role of social support and rural-urban differences in shaping these associations remains unclear. Using data from the National Study of Caregiving (NSOC, N = 1,422), this study applies moderated mediation analysis to examine whether dementia caregiving predicts depressive symptoms via interrupted sleep and whether social support moderates this relationship. In addition, separate analyses were conducted for rural and urban caregivers to assess contextual differences. Results indicate that dementia caregivers report significantly more sleep disturbances than non-dementia caregivers, which in turn contribute to higher levels of depression. Social support moderates the relationship between sleep disturbances and depression, with dementia caregivers who receive greater support exhibiting a stronger indirect effect of caregiving on depression through sleep disruptions compared to non-dementia caregivers. However, separate rural-urban analyses revealed that these effects were present only among urban caregivers. Among rural caregivers, dementia caregiving was not significantly associated with sleep disturbances or depression, suggesting that traditional stress and coping models may not fully capture the experiences of rural caregivers. These findings underscore the need for interventions targeting sleep health and social support among dementia caregivers, particularly in urban settings. Also, alternative frameworks may be necessary to address the unique challenges faced by rural caregivers. Understanding these contextual differences is crucial for developing targeted policies and support systems to improve caregiver mental health and well-being.

